# Intraindividual validation of 4D flow measurement against 2D flow measurements in aortas with bicuspid or tricuspid valves by cardiovascular magnetic resonance (CMR)

**DOI:** 10.1186/1532-429X-17-S1-P216

**Published:** 2015-02-03

**Authors:** Ahmed E Kharabish, Kristina Belker, Christian Meierhofer, Stefan Martinoff, Peter Ewert, Heiko Stern, Sohrab Fratz

**Affiliations:** Pediatric Cardiology and Congenital Heart Defects, German Heart center, Munich, Germany; Radiology Department, Cairo University, Cairo, Egypt; Radiology Department, German Heart Center, Munich, Germany

## Background

Theoretically, blood flow in the ascending aorta of patients with helical flow patterns, as in patients with bicuspid aortic valves (BAV), are underestimated by routine two dimensional (2D) phase contrast velocity encoding (PC-VENC). Four dimensional (4D) PC-VENC is theoretically not influenced by flow patterns. Hence, both, 2D PC-VENC and 4D PC VENC should result in similar blood flow measurements in the ascending aorta of subjects without helical flow patterns, as in subjects with tricuspid aortic valves (TAV).

## Methods

To test this hypothesis, we determined blood flow in the ascending aorta of sixteen patients with BAV and helical flow and eighteen healthy subjects with TAV and non-helical flow by 2D PC-VENC and 4D PC-VENC. Each data set was analyzed by two observers blinded to the other results.

## Results

In patients with BAV and helical flow, 4D PC-VENC resulted in systematically higher blood flow volumes than 2D PC-VENC. In subjects with TAV and non-helical flow, there was no systematical difference between 4D and 2D PC-VENC.

## Conclusions

Helical flow patterns as in the ascending aorta of patients with BAV may be more correctly quantified by 4D-VENC compared to 2D PC-VENC.

## Funding

All authors have no conflict of interest.

**Figure 1:**
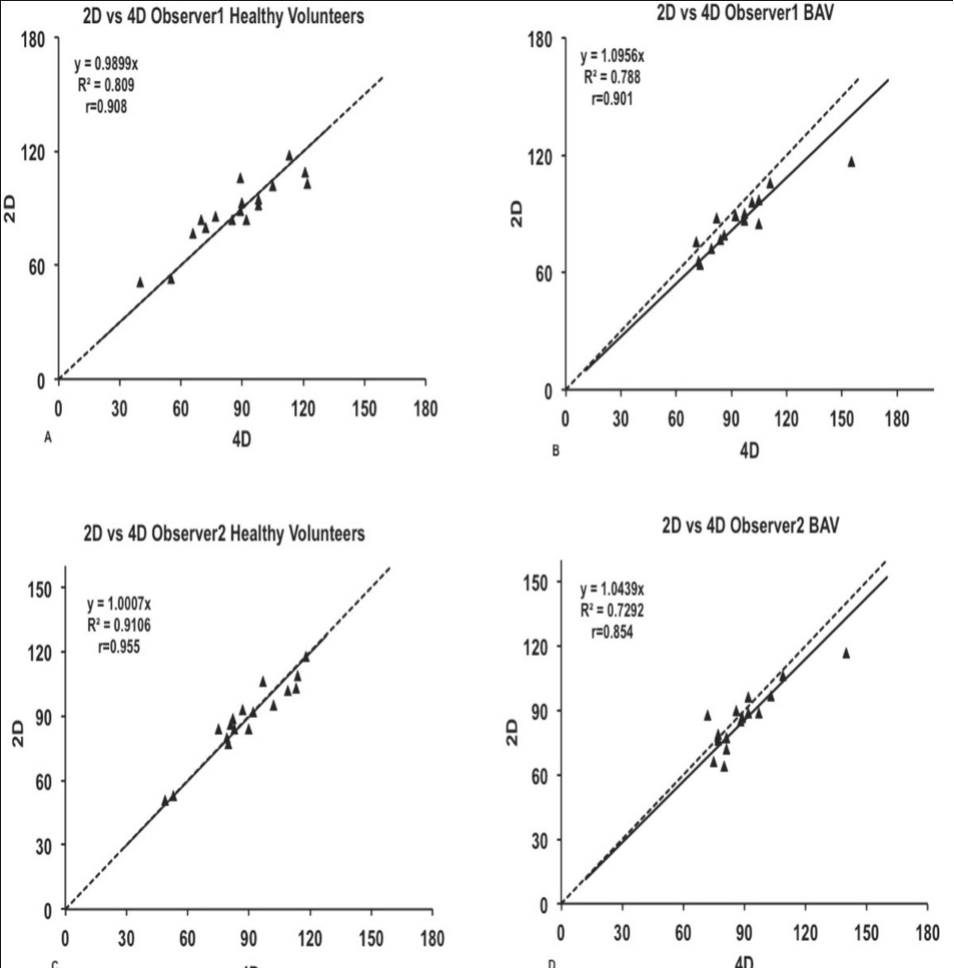
**Linear regression correlation.** Net flow values (ml) in the ascending aorta of both techniques by the two observers are plotted on both X-axis and Y-axis. The X-axis represents the four-dimensional (4D) method of flow analysis while the Y-axis represents the two-dimensional (2D) method of flow analysis in all four graphs. Graph A, and C represent Health volunteers, B and D represent patients with bicuspid aortic valve (BAV). A line of identity (dashed) y=x is added. The regression line (solid) indicates the offset between the two methods.

